# Activin/Nodal/TGF-β Pathway Inhibitor Accelerates BMP4-Induced Cochlear Gap Junction Formation During *in vitro* Differentiation of Embryonic Stem Cells

**DOI:** 10.3389/fcell.2021.602197

**Published:** 2021-04-21

**Authors:** Ichiro Fukunaga, Yoko Oe, Cheng Chen, Keiko Danzaki, Sayaka Ohta, Akito Koike, Katsuhisa Ikeda, Kazusaku Kamiya

**Affiliations:** Department of Otorhinolaryngology, Juntendo University Faculty of Medicine, Tokyo, Japan

**Keywords:** **embryonic** stem cells, SB431542, gap junction beta 2 gene, connexin 26 and 30, gap junction plaques, cochlear supporting cells

## Abstract

Mutations in gap junction beta-2 (*GJB2*), the gene that encodes connexin 26 (CX26), are the most frequent cause of hereditary deafness worldwide. We recently developed an *in vitro* model of *GJB*2-related deafness (induced CX26 gap junction-forming cells; iCX26GJCs) from mouse induced pluripotent stem cells (iPSCs) by using Bone morphogenetic protein 4 (BMP4) signaling-based floating cultures (serum-free culture of embryoid body-like aggregates with quick aggregation cultures; hereafter, SFEBq cultures) and adherent cultures. However, to use these cells as a disease model platform for high-throughput drug screening or regenerative therapy, cell yields must be substantially increased. In addition to BMP4, other factors may also induce CX26 gap junction formation. In the SFEBq cultures, the combination of BMP4 and the Activin/Nodal/TGF-β pathway inhibitor SB431542 (SB) resulted in greater production of isolatable CX26-expressing cell mass (CX26^+^ vesicles) and higher *Gjb2* mRNA levels than BMP4 treatment alone, suggesting that SB may promote BMP4-mediated production of CX26^+^ vesicles in a dose-dependent manner, thereby increasing the yield of highly purified iCX26GJCs. This is the first study to demonstrate that SB accelerates BMP4-induced iCX26GJC differentiation during stem cell floating culture. By controlling the concentration of SB supplementation in combination with CX26^+^ vesicle purification, large-scale production of highly purified iCX26GJCs suitable for high-throughput drug screening or regenerative therapy for *GJB2*-related deafness may be possible.

## Introduction

Hearing loss is the most common congenital sensory impairment worldwide (Chan et al., 2010). Approximately 1 child in 1,000 is born with severe or profound hearing loss or will develop hearing loss during early childhood (Morton, 1991; Petersen and Willems, 2006), and about half of such cases are attributable to genetic causes (Birkenhager et al., 2010). To date, there are >120 known forms of non-syndromic deafness associated with identified genetic loci^1^, and the types of cells associated with the disease are diverse. In particular, the gene gap junction beta-2 (*GJB2*), which encodes connexin (CX)26 protein, is the most common causative gene for non-syndromic sensorineural hearing loss (Rabionet et al., 2000; Morton and Nance, 2006). CX26 is expressed in non-sensory cochlear supporting cells and in such cochlear structures as the spiral limbus, stria vascularis, and spiral ligament (Kikuchi et al., 1995; Ahmad et al., 2003; Forge et al., 2003; Zhao and Yu, 2006; Liu and Zhao, 2008; Wingard and Zhao, 2015). CX26 and CX30 (encoded by *GJB6*) form functional heteromeric and heterotypic gap junction (GJ) channels in the cochlea (Sun et al., 2005). At the plasma membrane, GJs further assemble into semi-crystalline arrays known as gap junction plaques (GJPs) containing tens to thousands of GJs (Koval, 2006). GJs facilitate the rapid removal of K^+^ from the base of cochlear hair cells, resulting in cycling of K^+^ back into the endolymph of the cochlea to maintain cochlear homeostasis (Kikuchi et al., 2000). We previously showed that disruption of CX26-GJPs is associated with *Gjb2*-related hearing-loss pathogenesis and that assembly of cochlear GJPs is dependent on CX26 (Kamiya et al., 2014). Furthermore, we recently described the generation of mouse induced pluripotent stem cell (iPSC)-derived functional CX26 GJ-forming cells (induced CX26 GJ-forming cells, iCX26GJCs), as are found among the cochlea supporting cells, based on floating culture (serum-free floating culture of embryoid body-like aggregates with quick reaggregation, SFEBq culture) and adherent culture (Fukunaga et al., 2016) systems. The inner ear (cochlea) is an organ surrounded by bones and it is difficult to access from the outside. In addition, the inside of the cochlea is filled with lymph, and invasive procedures such as biopsy can lead to irreversible hearing loss. Accordingly, the inner ear is much more difficult to treat using human cells and tissues than in other sensory organs (eye, nose, tongue), and research on the pathophysiology and the development of treatment methods has been delayed. For this reason, rodents (mainly mouse) are a powerful tool for researching hearing loss. Of course, it is difficult to directly translate the results of drug screening of mouse iCX26GJC into human therapies. However, using mouse iCX26GJC for drug screening and conducting mouse experiments based on the results will be an important discovery opportunity for future applications to human deafness. However, before these cells can be used as a disease model for drug screening or for other large-scale assays, the cell culture system must be improved to increase the number of cells available at a single time. Our previous research suggested that the CX26-expressing cell masses (CX26^+^ vesicles) observed in day 7 aggregates that form as a result of BMP4 signaling in SFEBq cultures represent the origin of iCX26GJCs in the adherent culture (Fukunaga et al., 2016). If CX26^+^ vesicles in SFEBq cultures from embryonic stem cells (ESCs) or iPSCs could be obtained in a substantial quantity, we may have an adequate number of iCX26GJCs in adherent cultures. The inner ear, which is our target, is derived from the otic placode, which is part of the non-neural ectoderm (Barald and Kelley, 2004; Freter et al., 2008; Groves and Fekete, 2012). Several strategies to induce the differentiation of inner ear cells have been based on the generation of non-neural ectoderm from ESCs/iPSCs, which is promoted by the addition of BMP4, TGF-β inhibitor, and wnt inhibitor (Koehler et al., 2013; Ronaghi et al., 2014; Ealy et al., 2016). BMP4 is a strong neuronal inhibitor (Schuldiner et al., 2000; Tropepe et al., 2001; Munoz-Sanjuan and Brivanlou, 2002) and acts as a potent mesoderm induction factor (Wiles and Johansson, 1999; Czyz and Wobus, 2001) in stem cell differentiation. It has been reported that BMP promotes the differentiation of CX43-expressing cells such as astrocytes (Bani-Yaghoub et al., 2000) and cardiomyocytes (Takei et al., 2009). Similarly, the Activin/Nodal/TGF-β pathway inhibitor SB431542 (SB) has been implicated in efficient neural conversion of ESCs and iPSCs via inhibition of SMAD signaling (Chambers et al., 2009; Chambers et al., 2012) and by blocking the progression of stem cell differentiation toward trophectoderm, mesoderm, and endoderm lineages (Li et al., 2013). However, we did not find any reports that SB promotes the differentiation of stem cells into CX-expressing cells. Given this background, we hypothesized that SB may affect the differentiation of iCX26GJCs. At the beginning of the experiment, we compared the drug responsiveness between ESCs and iPSCs using CX26^+^ vesicles as an indicator. As a result, ESC was more responsive to drugs than iPSC (Supplementary Figures 1A–D). Therefore, in the present study, we evaluated SFEBq culture conditions incorporating BMP4 and/or SB with the aim of generating iCX26GJCs from mouse ESCs at a greater efficiency than those generated from iPSCs.

## Materials and Methods

### ESC Culture

The mouse ESC line (EB5 cells; Niwa et al., 2002; Ogawa et al., 2004) was provided by the RIKEN Bio Resource Center Cell Bank and maintained under feeder-free conditions with 2i-LIF medium as described (Ying et al., 2008). Briefly, ESCs were maintained on gelatin-containing N2B27 medium consisting of a 1:1 (v/v) mixture of Advanced DMEM/F12 and neurobasal medium (Invitrogen) supplemented with 1 mM GlutaMAX (Invitrogen), 1% N2 supplement (Invitrogen), 2% B27 supplement (Invitrogen), 3 μM CHIR99021 (Stemgent), 1 μM PD0325901 (Santa Cruz), and 1,000 U ml^–1^ of leukemia inhibitory factor (Millipore).

### Differentiation of ESCs

**FIGURE 1:**
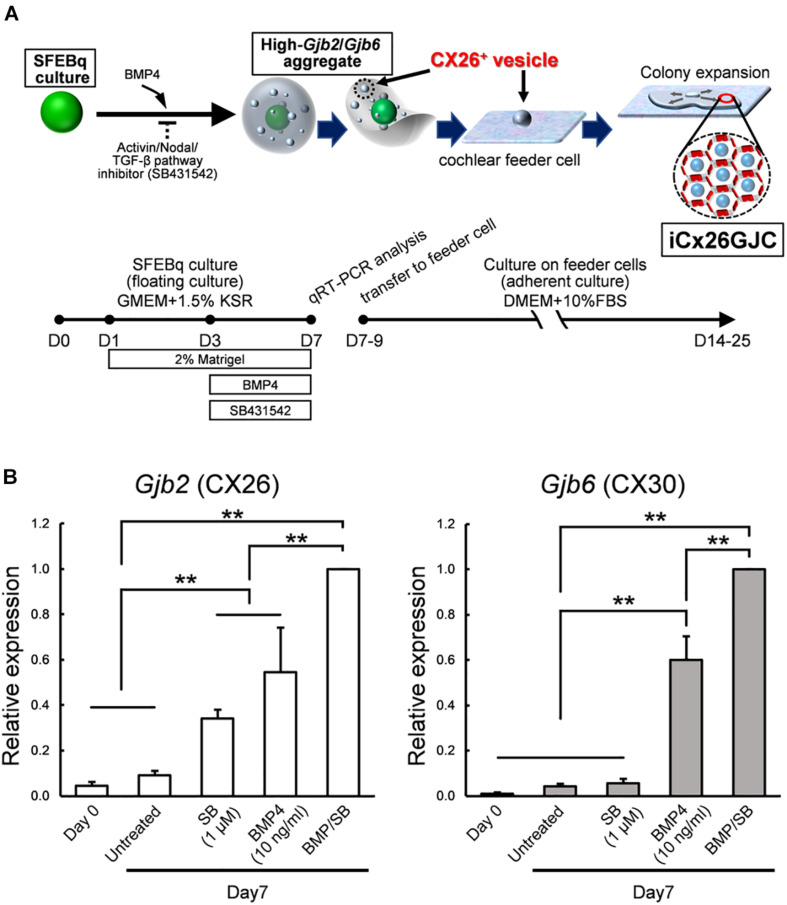
Culture conditions for cells that expressed high levels of *Gjb2* (CX26) and *Gjb6* (CX30) mRNA. **(A)** A schematic procedure for differentiating iCx26GJCs from mouse ESCs. SFEBq, serum-free floating culture of embryoid body-like aggregates with quick reaggregation; KSR, knockout serum replacement; BMP4, Bone morphogenetic protein 4; SB431542, Activin/Nodal/TGF-β pathway inhibitor. **(B)** Relative expression of mRNA at day 0 (for undifferentiated ESCs) and at day 7 for untreated, BMP4-treated, SB-treated, and BMP4/SB-treated aggregates. mRNA expression levels were normalized to those of BMP4 cultures on day 7. The data are expressed as the mean ± SE from five independently generated cell cultures per treatment; for each replicate, expression was assessed in eight aggregates per treatment. Differences among samples were assessed by one-way ANOVA and Scheffe’s multiple comparison test; **p < 0.01.

Induction of iCX26GJCs was performed as shown in Figure 1A. Briefly, ESCs were dissociated with Accutase (Innovative Cell Technologies, Inc.); suspended in differentiation medium (G-MEM, Gibco) supplemented with 1.5% (v/v) knockout serum replacement (Gibco), 0.1 mM nonessential amino acids (Gibco), 1 mM sodium pyruvate (Gibco), and 0.1 mM 2-mercaptoethanol; and then plated at 100 μl/well (3,000 cells) in 96-well low-cell-attachment V-bottom plates (Sumitomo Bakelite). Recombinant BMP4 (obtained from Miltenyi Biotec) was diluted with DW at 100 μg/ml, and SB431542 (obtained from Tocris Bioscience) was diluted with DMSO at 10 mM. On day 1, half of the medium (50 μl) in each well was replaced with fresh differentiation medium containing 4% (v/v) Matrigel (BD Bioscience). On day 3, one of three types of media was added to the culture: medium containing BMP4 (10 ng/ml, final concentration), SB (1, 5, or 10 μM, final concentration), or both factors at the aforementioned concentrations. BMP4 and SB stock solutions were prepared at a 5 × concentration in fresh medium. Stock solutions were stored for up to 6 months at -20°C. On days 7–11, the aggregates were partially dissected, and the CX26^+^ vesicles (20–60 μm) were mechanically isolated under stereo microscope and collected using forceps. The CX26^+^ vesicles were transferred to adherent cultures containing trypsin-resistant inner-ear cells (TRICs) in the growth medium, which consisted of Dulbecco’s modified Eagle’s medium (DMEM) GlutaMAX (Gibco) and 10% (w/v) fetal bovine serum (FBS).

TRICs were isolated by exposing cochlear tissue to trypsin and screening for trypsin-resistant cells. The cochlear tissue (from 10-week-old mice, obtained from CLEA Japan, Inc.) used for the preparation of TRICs included the organ of Corti, basilar membrane, and lateral wall and mainly comprised supporting cells, hair cells, cochlear fibrocytes, and other cells in the basilar membrane. This cell line was used as inner ear-derived feeder cells on which to proliferate the otic progenitor cells. For the feeder cell layer preparation, 3 × 10^5^ TRICs/cm^2^ were seeded onto gelatin-coated wells of 24-well culture plates and mitomycin C (10 mg/ml) treatment for 3 h.

### Analysis of *Gjb2* and *Gjb6* mRNA Expression

Total RNA was isolated from day 7 aggregates using reagents from an RNeasy Plus Mini kit (Qiagen) and reverse transcribed into cDNA using reagents from a Prime Script II first strand cDNA synthesis kit (Takara). Real-time PCR was performed with the reverse transcription products, TaqMan Fast Advanced Master Mix reagents (Applied Biosystems), and a gene-specific TaqMan Probe (see below; Applied Biosystems) on a StepOne Real-Time PCR system (Applied Biosystems). Each sample was run in triplicate. Applied Biosystems StepOne software was used to analyze the Ct values of the different mRNAs normalized to expression of the endogenous control, *Actb* mRNA. TaqMan Probes (Assay ID; Applied Biosystems) were used to detect the expression of mouse *Gjb2* (Mm00433643_s1), *Gjb6* (Mm00433661_s1), and *Actb* mRNAs (Mm02619580_g1).

### Immunostaining and Image Acquisition

Aggregates were fixed with 4% (w/v) paraformaldehyde in 0.01 M phosphate-buffered saline (PBS) for 1 h at room temperature. For whole mounts, the aggregates were permeabilized with 0.5% (w/v) Triton X-100 (Sigma-Aldrich) in 0.01 M PBS for 30 min. Then, the samples were washed twice with 0.01 M PBS and blocked with 2% (w/v) bovine serum albumin in 0.01 M PBS for 30 min. Cells from adherent cultures were fixed with 4% (w/v) paraformaldehyde in 0.01 M PBS for 15 min at room temperature and then were permeabilized with 0.5% (w/v) Triton X-100 in 0.01 M PBS for 5 min. Samples were washed twice with 0.01 M PBS and blocked with 2% (w/v) bovine serum albumin in 0.01 M PBS for 30 min. For immunofluorescence staining, 1% (w/v) bovine serum albumin in 0.01 M PBS was used to dilute the primary and secondary antibody solutions. Each sample was incubated in a primary antibody solution—CX26 (1:150, mouse IgG, 33-5800, Invitrogen), CX30 (1:300, rabbit IgG, 71-2200, Invitrogen), PAX2 (1:100, rabbit IgG, 71-6000, Invitrogen), PAX8 (1:100, rabbit IgG, ab97477, Abcam), or E-Cadherin (1:100, rabbit IgG, 3195S, Cell Signaling)—for 1 h after blocking. The secondary antibodies were Alexa Fluor 594–conjugated anti-mouse IgG (A11032, Invitrogen), Alexa Fluor 488–conjugated anti-rabbit IgG (A11070, Invitrogen), and phalloidin FITC (A12379, Invitrogen). Samples were washed twice with 0.01 M PBS and mounted with mounting medium (VECTASHIELD Mounting Medium with DAPI, Vector). Fluorescence confocal images were obtained with an LSM510-META confocal microscope (Zeiss). Z-stacks of images were collected at 0.5-μm intervals, and the single-image stacks were constructed using LSM Image Browser (Zeiss). Three-dimensional images were constructed with z-stacked confocal images using IMARIS (Bitplane).

### FACS Analysis

Cells were counted by FACSCalibur (BD Biosciences) and the data analyzed with FlowJo software (BD Biosciences). For cell preparation, cells were dissociated to single cells by 0.25% trypsin-EDTA treatment, fixed in 4% paraformaldehyde in DPBS at 4°C, and permeabilized in 0.1% Triton in DPBS at 4°C. Primary antibody (CX26, 1:150, mouse IgG, Invitrogen) was incubated at RT for 1 hr. Secondary antibodies (Alexa Fluor 488–conjugated anti Mouse, 1:1,000, Invitrogen) was incubated at RT for 1 h. Cells were washed with DPBS and counted using FACS.

### Statistical Analyses

The data were analyzed using Microsoft Excel software and are presented as the mean ± SE. A two-tailed Student’s *t*-test, with a significance criterion of *p* < 0.05, was used to compare the GJP lengths. One-way ANOVA and Scheffe’s multiple comparison test, with a significance criterion of *p* < 0.05, were used to compare *Gjb2* and *Gjb6* mRNA levels and the number of CX26^+^ vesicles.

## Results

### SB Promoted BMP4-Induced *Gjb2/Gjb6* mRNA Expression in SFEBq Cultures

iCX26GJCs were induced from mouse ESCs as described (Fukunaga et al., 2016), and the conditions required for differentiation were then assessed. ESCs were cultured in SFEBq medium containing BMP4, SB, or BMP4 plus SB. Aggregates were collected on day 7, and mRNA (*Gjb2* and *Gjb6*) levels under different culture treatments were measured. Cultures treated with BMP4 and BMP4/SB produced more *Gjb2/Gjb6* mRNA than cultures treated with SB alone or control cultures (Figure 1B). BMP4 induces *Gjb2*/*Gjb6* mRNA expression during iPSC differentiation (Fukunaga et al., 2016). In addition, ESCs cultured in differentiation medium supplemented with BMP4 and SB showed greater expression levels of mRNA (*Gjb2*, 1.8-fold greater; *Gjb6*, 1.7-fold greater) as compared with those cultured with BMP4 alone.

### SB Promoted Formation of CX26-Expressing Small Vesicles in SFEBq Cultures

**FIGURE 2:**
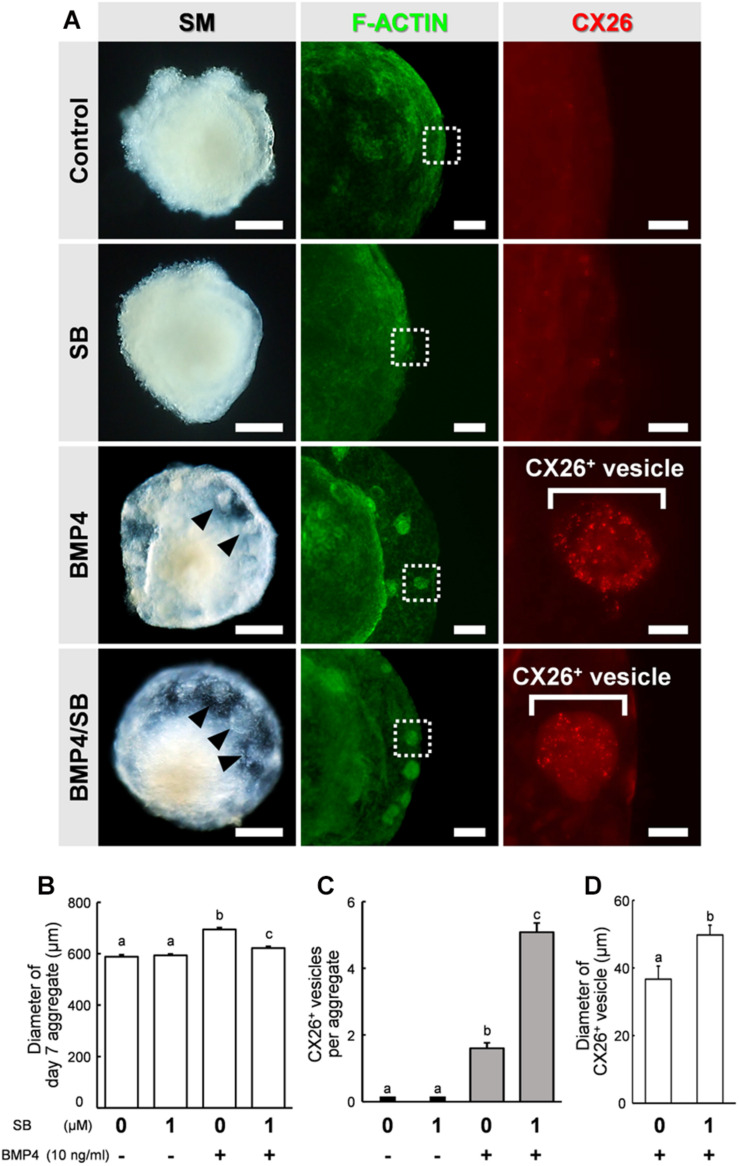
Stereomicroscopic images and immunostained aggregates derived from ESCs after 7 days in SFEBq cultures. **(A)** Left column: stereomicroscopic (SM) images of cells on day 7. Middle column: cells immunostained for F-actin (green). Right column: higher-magnification image of the boxed regions in the middle columns, showing immunostaining for CX26 (red). Arrowheads point to small vesicles. Scale bars, 200 μm (left column); 100 μm (middle column); 20 μm (right column). **(B)** Average diameter of day 7 aggregate (*n* = 15–32 aggregates from two to four independent experiments). **(C)** The average number of CX26^+^ vesicles per aggregate (*n* = 24 aggregates from three independent experiments). **(D)** Average diameter of CX26^+^ vesicles in day 7 aggregate (*n* = 12–24 CX26^+^ vesicles from three independent experiments). The data are expressed as the mean ± SE. Statistical differences among treatments were assessed by one-way ANOVA and Scheffe’s multiple comparison test, or Student’s *t*-test. Different letters (a–c) represent significant differences, *p* < 0.01.

By day 7 of differentiation, the aggregates showed differentiated outer regions with a morphology similar to that reported previously (Fukunaga et al., 2016). Clear outer epithelia and small vesicles were observed beneath the outer epithelium of BMP4 alone or BMP4/SB-treated cells. By contrast, no small vesicles were observed for the control or SB-treated cells (Figure 2A, left column). To determine the location of CX26 in the cell aggregates, immunohistochemistry was performed. In BMP4- or BMP4/SB-treated aggregates, CX26^+^ vesicles were observed (Figure 2A, right column).

The aggregates were collected, and the diameter of the day 7 aggregate, the number of CX26^+^ vesicles, and the diameter of CX26^+^ vesicles were compared among the different treatment groups (Figures 2B–D). The diameter of the day 7 aggregate cultured under different conditions were Control (mean ± SE, 588.1 ± 7.60 μm), SB (mean ± SE, 593.4 ± 5.61 μm), BMP4 (mean ± SE, 694.1 ± 7.40 μm), and BMP4 / SB (mean ± SE, 622.0 ± 6.53 μm), and the aggregate treated with BMP4 alone was the largest. Cells treated with BMP4/SB had more CX26^+^ vesicles (mean ± SE, 5.08 ± 0.28 CX26^+^ vesicles per aggregate) than those cultured with BMP4 alone (mean ± SE, 1.6 ± 0.16 CX26^+^ vesicles per aggregate). Furthermore, when measuring the diameter of the CX26^+^ vesicles formed on the day 7 aggregate, the aggregates treated with BMP4/SB formed larger CX26^+^ vesicles (mean ± SE, 49.7 ± 3.36 μm) than the aggregates treated only with BMP4 (mean ± SE, 36.7 ± 2.93 μm).

**FIGURE 3:**
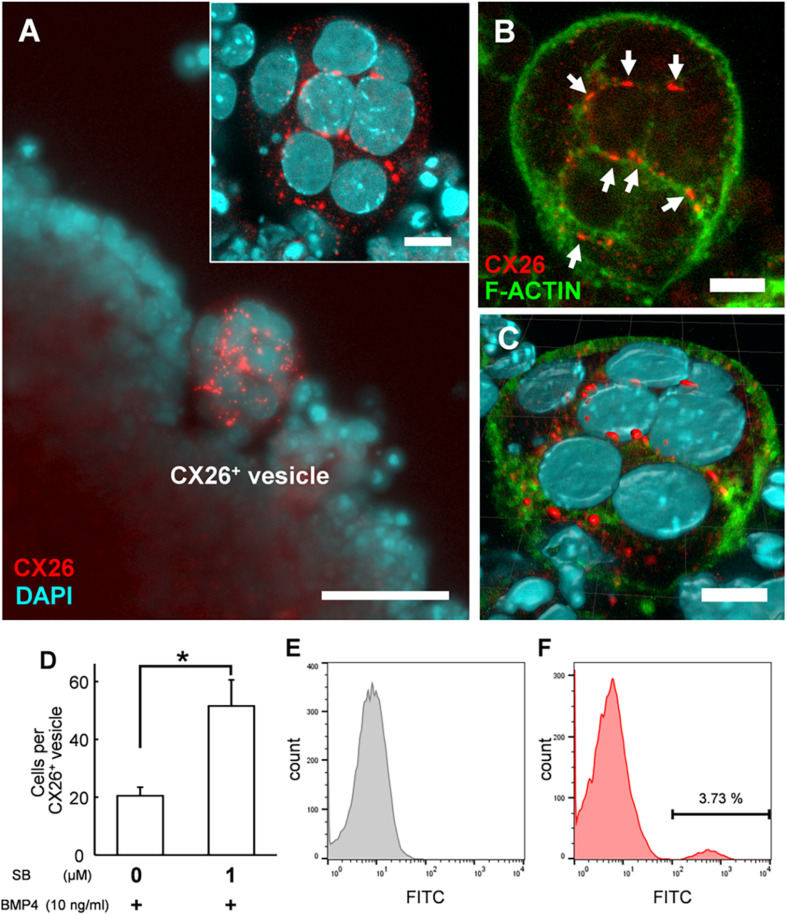
Confocal images of CX26^+^ vesicles in BMP4/SB-treated ESC aggregates. **(A)** Merged image of CX26-immunostained (red) and DAPI-stained (blue) cells in CX26^+^ vesicles. Inset in **(A)** Higher-magnification image of the CX26^+^ vesicle from this aggregate. **(B)** Merged images of CX26-immunostained (red) and F-actin-stained (green) cells in the same vesicle as shown in **(A)**. Arrows point to GJPs. **(C)** The three-dimensional image reconstructed from the same CX26^+^ vesicle. **(D)** The average number of cells that composed CX26^+^ vesicle (n = 12–18 CX26^+^ vesicles from three independent experiments). Statistical differences among treatments were assessed by Student’s *t*-test; *p < 0.05. **(E,F)** FACS analysis for CX26^+^ populations in day 7 aggregate. **(E)** Gray, control (negative control); **(F)** Red, Numbers in the histogram indicate the percentage of positive cells. Scale bars: 50 μm **(A)**, 10 μm (inset in **A–C**).

**FIGURE 4:**
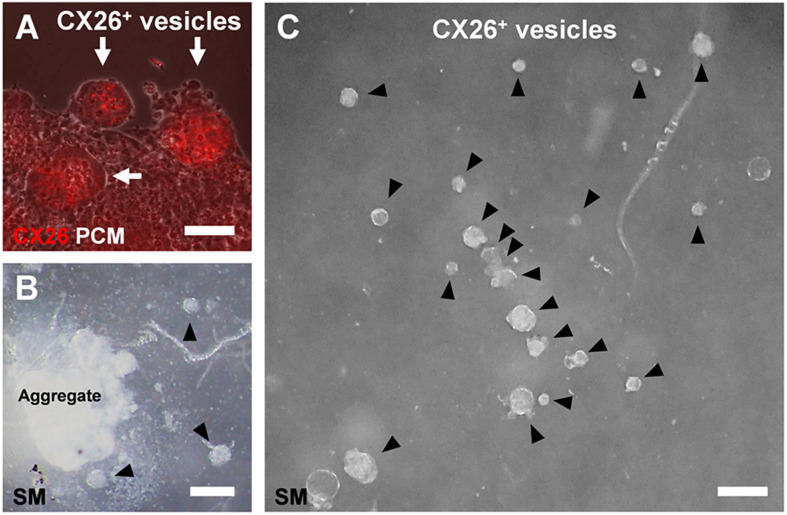
CX26^+^ vesicles in aggregates with BMP4 and SB supplementation. **(A)** Merged images from fluorescence microscopy of CX26 immunostaining (red) and phase-contrast microscopy (PCM, white) of ESC aggregates at day 7. Arrows point to CX26^+^ vesicles containing CX26GJCs. **(B,C)** Stereomicroscopic (SM) images of isolated CX26^+^ vesicles from ESC aggregates at day 7. Arrowheads point to CX26^+^ vesicles. CX26^+^ vesicles were easily isolated from partially dissected ESC aggregates **(B)** and then were mechanically collected **(C)**. Scale bars: 50 μm **(A)**, 100 μm **(B,C)**.

In the confocal analysis of the day 7 aggregates from BMP4/SB-treated cells, CX26-expressing cells were dispersed throughout the numerous CX26^+^ vesicles (Figure 3A and Supplementary Video 1). These cells formed CX26^+^ GJs at their cell-cell borders (Figure 3B). In the three-dimensional construction of the confocal images, we observed large planar CX26-containing GJPs (Figure 3C and Supplementary Video 2), which, as we reported previously (Kamiya et al., 2014; Fukunaga et al., 2016), are characteristic of the mouse cochlea. On the other hand, when counting the number of CX26^+^ cells that composed CX26^+^ vesicle, the aggregate treated with BMP4/SB consisted of more CX26^+^ cells (mean ± SE, 51.6 ± 9.0 cells per CX26^+^ vesicle) than the aggregate cultured only with BMP4 (mean ± SE, 20.5 ± 2.9 cells per CX26^+^ vesicle; Figure 3D). In addition, the positive rate of CX26^+^ cells in the day 7 aggregate treated with BMP/SB was 3.73 % (Figures 3E,F). CX26^+^ vesicles were found to exist separately from core region in BMP4 alone or BMP4/SB-treated aggregates (Figures 4A,B), suggesting that they could be easily isolated. Numerous CX26^+^ vesicles were mechanically collected as a purified iCX26GJC population (Figure 4C).

### ESC-Derived iCX26GJCs That Co-expressed CX30 in Adherent Cultures Formed Gap Junctions

**FIGURE 5:**
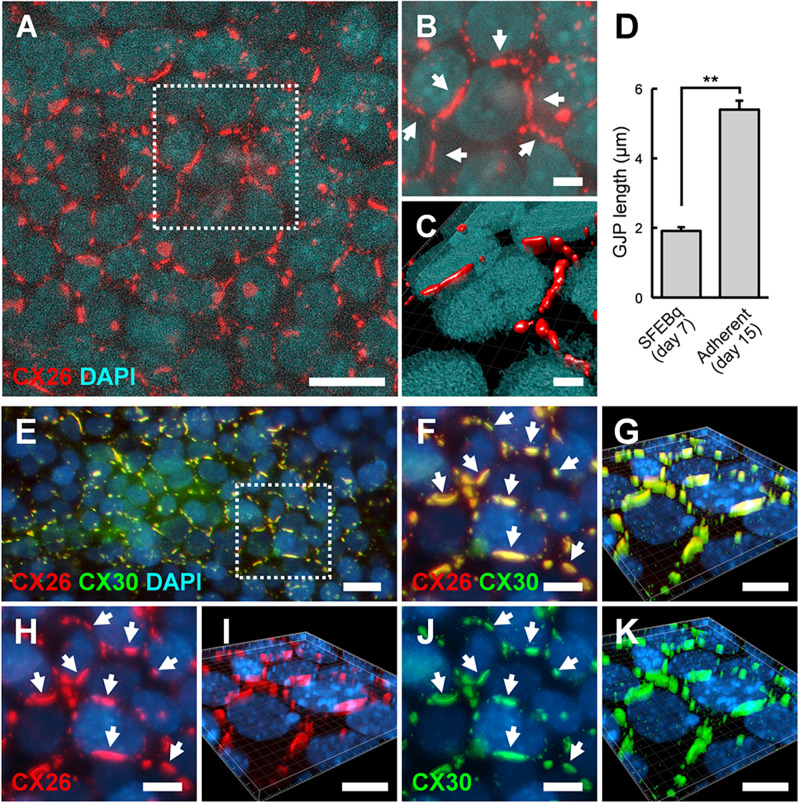
ESC-derived CX26GJCs formed large CX26GJPs and CX26/CX30-containing GJPs. **(A)** Merged images of CX26-immunostained (red) and DAPI-stained (blue) cells from an adherent culture at day 15. **(B)** Higher-magnification image of the boxed region in **(A)**. Arrows point to GJPs. **(C)** The three-dimensional image was reconstructed from the same region as shown in **(B)**. **(D)** Mean lengths of the longest dimension of the GJPs along a single cell border in SFEBq cultures (SFEBq) at day 7 and adherent cultures (Adherent) at day 15 (SFEBq culture, *n* = 43 cell borders from 5 aggregates; adherent culture, *n* = 41 cell borders from 4 wells). For assessments, the procedures were repeated three times to generate cells. Statistical differences between samples were assessed by Student’s *t*-test; **p < 0.01. The data are expressed as the mean ± SE. **(E)** Merged images of CX26-immunostained (red), CX30-immunostained (green), and DAPI-stained (blue) cells from adherent cultures at day 15. **(F,H,J)** Higher-magnification image of the boxed region in **(E)**. Staining for CX26 (red), CX30 (green), and DAPI (blue). Arrows point to the large GJPs. **(G,I,K)** The three-dimensional images were reconstructed from **(F,H,J)**, respectively. Arrows point to the large GJPs. Scale bars: 20 μm **(A)**, 10 μm **(E–K)**, 5 μm **(B)**, and 3 μm **(C)**.

Between day 7 and 9, BMP4/SB-treated aggregates were transferred onto cochlear-derived feeder cells, namely TRICs, as follows. The differentiated regions with CX26^+^ vesicles were separated from the day 7 aggregates and subcultured in DMEM GlutaMAX with 10% (v/v) FBS on TRIC feeder cells. The subcultured regions containing CX26^+^ vesicles colonized the TRIC feeder cells. In the adherent cultures at day 10 (3 days after transferred onto TRIC feeder cells), CX26^+^ vesicle derived colony co-expressed CX26, Pax2, PAX8, and E-cadherin (Supplementary Figure 1). In the adherent cultures at day 15 (8 days after transferred), CX26-containing GJPs were observed (Figures 5A–C), as found in cochlear supporting cells (Kamiya et al., 2014; Fukunaga et al., 2016). The mean length of the longest dimension of the GJPs along a single cell border was 1.91 ± 0.11 μm for BMP4/SB-treated on day 7 aggregates, which increased significantly to 5.39 ± 0.25 μm in the adherent cultures at day 15 on TRIC feeder cells (Figure 5D), similar to observations when iPSCs were used (Fukunaga et al., 2016). To assess the similarities between these cells and cochlear cells, we characterized the expression of CX30, which is frequently absent in hereditary deafness. CX30 co-localized with CX26 in most CX26-GJPs in the differentiated cells (Figures 5E–K and Supplementary Video 3), suggesting that CX26 and CX30 were the two main components of these GJPs, as was found for cochlear cells (Kamiya et al., 2014; Fukunaga et al., 2016). In addition, the scrape loading-dye transfer assay revealed that mouse ESC-derived iCX26GJC forms functional GJs (Supplementary Figure 3) as in our previous report (Fukunaga et al., 2016).

### SB Addition Increased the Number of CX26^+^ Vesicles in a Dose-Dependent Manner

**FIGURE 6:**
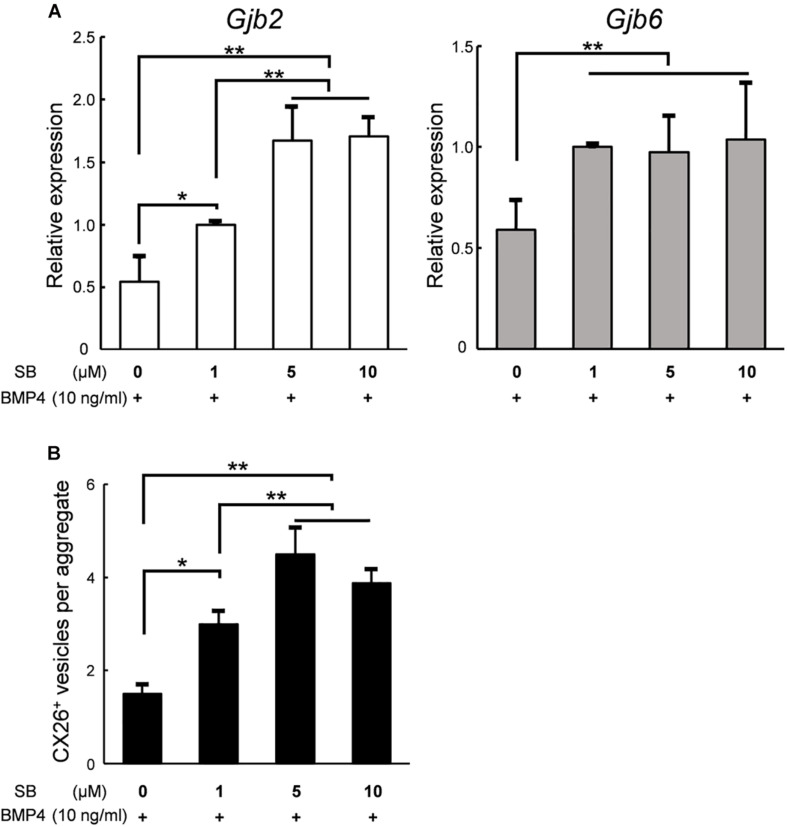
In SFEBq cultures, SB had a dose-dependent effect on *Gjb2*/*Gjb6* mRNA expression and the number of CX26^+^ vesicles. **(A)** Relative expression of *Gjb2* and *Gjb6* mRNA in day 7 aggregates from SFEBq cultures after treatment with BMP4 (10 ng/ml) alone or with SB (1–10 μM) as indicated. mRNA expression was normalized to that of cultures treated with BMP4/1 μM SB. The data are expressed as the mean ± SE from five independently generated cell cultures per treatment; for each replicate, expression was assessed in eight aggregates per treatment. **(B)** The average number of CX26^+^ vesicles per aggregate from aggregates treated as described in **(A)**. The data are expressed as the mean ± SE from three independently generated cell cultures per treatment; for each replicate, vesicles were quantified for 2–3 aggregates per treatment (*n* = 8 aggregates in total). Statistical differences among samples were assessed by a one-way ANOVA and Scheffe’s multiple comparison test; *p < 0.05; **p < 0.01.

Finally, to produce a large number of iCX26GJCs in SFEBq cultures, we examined whether the differentiation from ES cells to iCX26GJCs depended on the concentration of SB. Based on a quantitative reverse transcription-PCR (qRT-PCR) analysis, aggregates treated with BMP4 and either 5 or 10 μM SB showed higher expression of *Gjb2* mRNA (BMP4/5 μM SB, 1.7-fold greater; BMP4/10 μM SB, 1.7-fold greater) as compared with treatment with BMP4/1 μM SB. Expression of *Gjb6* was not affected by the concentration of SB (Figure 6A). In the SB alone group, expression of *Gjb2* and *Gjb6* was consistent across all three concentrations of SB (Supplementary Figure 3A). We next determined the number of CX26^+^ vesicles in day 7 aggregates based on immunostaining. Again, aggregates treated with BMP4 and either 5 or 10 μM SB showed a greater number of CX26^+^ vesicles (BMP4/5 μM SB, 1.5-fold greater; BMP4/10 μM SB, 1.3-fold greater) compared with BMP4/1 μM SB (Figure 6B). Conversely, in the SB alone group, there was no difference in the number of small vesicles across all three concentrations of SB (Supplementary Figure 1B).

## Discussion

**FIGURE 7:**
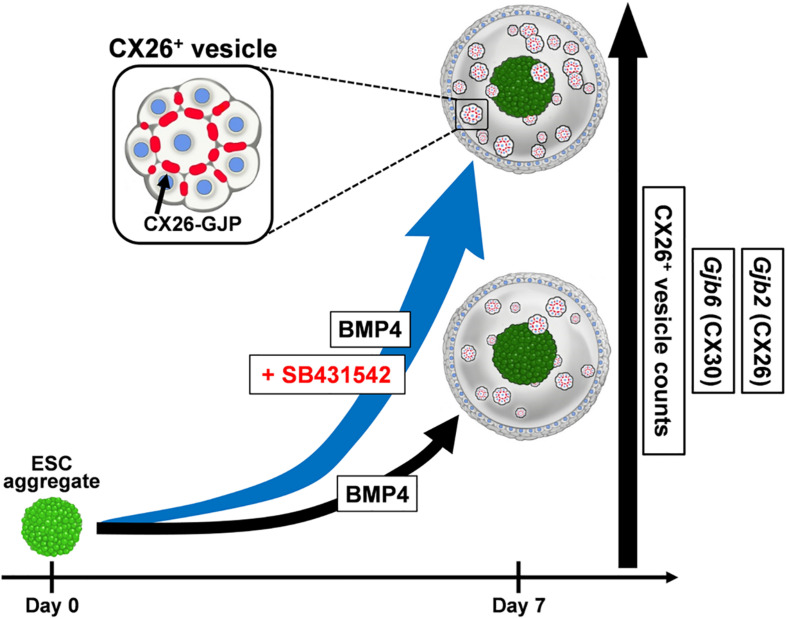
A schematic illustration of the effect of SB431542 on CX26 GJ formation in BMP4-induced ESC differentiation. In the BMP4-based inner-ear three-dimensional differentiation from ESCs, addition of SB431542 was associated with significantly higher mRNA levels of *Gjb2* (CX26) and *Gjb6* (CX30) and CX26^+^ vesicle counts relative to cultures without the addition of SB431542. SB431542 was demonstrated to be an accelerator of GJ formation.

ESC/iPSC-derived *in vitro* models can be a powerful platform for understanding pathological mechanisms and developing therapeutic methods. Previously, we produced CX26 gap junction-forming cells (iCX26GJCs), which have characteristics of cochlear supporting cells, from mouse iPSCs by using BMP4 signaling in combination with SFEBq cultures and subsequent adherent cultures (Fukunaga et al., 2016). In this study, we evaluated the necessary conditions for the differentiation of pluripotent stem cells using SFEBq cultures containing BMP4 and/or SB for large-scale production of iCX26GJCs. SB accelerated BMP4-induced iCX26GJC differentiation in the SFEBq cultures. The SFEBq culture system is currently the most suitable method for inducing neural ectoderm from ESCs/iPSCs, and it leads to the differentiation of these cells into various ectoderm-derived tissues, for example, forebrain, midbrain, hindbrain, optic cup, and otic cup, depending on the culture conditions (Wataya et al., 2008; Eiraku and Sasai, 2012; Koehler et al., 2017; Takata et al., 2017). In SFEBq cultures, BMP4 upregulates a non-neural ectoderm marker (*Dlx3*) and downregulates a neuroectoderm marker (*Sox1*; Koehler et al., 2013). In contrast, SB induces suppression of brachyury and induces expression of transcription factor activator protein 2 (AP2, also known as TFAP2), and it is thought to promote proper non-neural induction after BMP4 treatment (Koehler et al., 2013; Koehler and Hashino, 2014). The AP2 transcription factor regulates the expression of a variety of genes during development (Saffer et al., 1991; Nottoli et al., 1998), and, based on bioinformatic predictions, it is inferred that several transcription factors (including AP2) are likely to have a role in regulating the expression of *Gjb2* and *Gjb6* (Common et al., 2005; Jayalakshmi et al., 2019). Furthermore, *Gjb2* is upregulated by AP2 in normal tissue (Tu et al., 2001; Adam and Cyr, 2016). In SFEBq cultures, AP2 is used as a marker of non-neural ectoderm, and its expression is observed in epithelium-like structures formed outside of aggregates (Koehler et al., 2013, 2017). From these reports and our results, we speculate that the addition of SB to BMP-based SFEBq cultures increased the non-neural ectoderm region expressing AP2 in the aggregate (Koehler et al., 2013), resulting in increased *Gjb2*/*Gjb6* mRNA expression and production of CX26^+^ vesicles. That is, SB strongly promoted BMP4-mediated differentiation into iCX26GJCs in SFEBq cultures (Figure 7). On the other hand, in the modified SFEBq culture in this study, we confirmed that the activin/Nodal/TGF-β pathway was inhibited by the addition of SB431542 (Supplementary Figure 5), as in the previous study (Osakada et al., 2009).

In the adherent cultures at day 10 (3 days after transferred), iCX26GJC co-expressed PAX2/PAX8/E-cadherin (Supplementary Figure 1). In otic development, CX26 is observed in otic vesicles (Embryonic day (E) 10.5; Chan et al., 2010). At E9-11, PAX2, PAX8, and E-cadherin are observed in otic vesicles (Morton, 1991; Petersen and Willems, 2006; Birkenhager et al., 2010), and these combinations are used as a marker for otic vesicles for stem cell differentiation (Rabionet et al., 2000; Morton and Nance, 2006; Birkenhager et al., 2010). Furthermore, in the adherent culture at day 15 (8 days after transferred), iCX26GJCs co-expressed CX30 and formed GJPs (Figures 5E–K). In cochlear non-sensory regions including supporting cells, CX30 is co-expressed with CX26 and forms GJs (Kamiya et al., 2014; Fukunaga et al., 2016; Defourny et al., 2019). From our results and these reports, we suggested that proliferated iCX26GJC in the adherent culture derived from CX26^+^ vesicle is likely to have differentiated into cochlear supporting cells. Mechanical dissection of the aggregates (Figures 3B,C) suggested that CX26^+^ vesicles could be easily purified and isolated for large-scale production of iCX26GJCs after adherent culture on feeder cells (Figure 5).

In many reports for drug screening, 0.5–1.0 × 10^4^ Hela cells per well are seeded in 96 wells for use the next day. Assuming that it will be used after culturing for a certain period (7 days), 2 × 10^3^ Hela cells per well will be required (Ke et al., 2004). In this study, about 200–300 CX26^+^ cells per aggregate were confirmed. Since it takes at least 7 days to complete the induction to iCX26GJC, it is expected that 7–10 aggregates/well (10 × 200 = 2,000; 7 × 300 = 2,100) will be required when used for drug screening.

On the other hand, in this study, we separated the CX26+ vesicles from the aggregate by using forceps under microscopy. This step may be a drawback for large-scale cell production in the future. Cell separation techniques include physical methods (manual pipetting, density gradient centrifugation, cell adhesion) and affinity-based methods (FACS, MACS; Diogo et al., 2012). Recently, several methods have been reported for purifying target cells induced from ES/iPS cells. For example, pure retinal pigment epithelial cell sheets are produced by a combination of manually picking up and subculture of target cells (Iwasaki et al., 2016). In addition, it has been reported that corneal epithelial cells can be purified by a combination of MACS and differences in cell adhesion (Shibata et al., 2020). On the other hand, a method for purifying cardiomyocytes by density gradient centrifugation using percoll has been reported (Xu et al., 2002). In addition, purification methods by using culture media that are considered to be extremely unlikely to be intervened by human, have been reported (Tohyama et al., 2016). From these reports, it is considered necessary to study the conditions of purification methods that do not rely on manual picking up, such as density gradient centrifugation, difference in cell adhesion, and antibody used for FACS, as future tasks.

Increases in mRNA expression and CX26^+^ small vesicles were found to depend on the concentration of SB. However, with respect to the expression of *Gjb2* and the number of CX26^+^ vesicles, there was no significant difference between treatment with 5 or 10 μM SB (Figure 6). These results indicated that iCX26GJCs could be most efficiently induced with the BMP4/5 μM SB combination. These data suggest that SB promotes BMP4-mediated production of CX26^+^ vesicles in a dose-dependent manner, thereby increasing the yield of highly purified iCX26GJCs.

This is the first study to show that SB accelerates BMP4-induced *Gjb2*/*Gjb6* expression and CX26^+^ vesicle production during *in vitro* differentiation of ESCs (Figure 7). By controlling the concentration of SB in combination with CX26^+^ vesicle purification, large-scale production of highly purified iCX26GJCs for high-throughput screening of drugs that target *GJB2*-related deafness may be possible.

## Data Availability Statement

The original contributions presented in the study are included in the article/Supplementary Material, further inquiries can be directed to the corresponding author/s.

## Ethics Statement

The animal study was reviewed and approved by the Institutional Animal Care and Use Committee at Juntendo University School of Medicine.

## Author Contributions

KK: project administration and supervision. KK and KI: conceptualization. KK and IF: data curation, formal analysis, funding acquisition, investigation, methodology, visualization, writing—original draft preparation, and writing—review and editing. KK, IF, CC, YO, KD, SO, AK, and KI: resources. KK, IF, CC, YO, KD, SO, and AK: validation. All authors contributed to the article and approved the submitted version.

## Conflict of Interest

The authors declare that the research was conducted in the absence of any commercial or financial relationships that could be construed as a potential conflict of interest.

## Footnotes

1 http://hereditaryhearingloss.org
