# Supplementary material for: Activin/Nodal/TGF-β Pathway Inhibitor Accelerates BMP4-Induced Cochlear Gap Junction Formation During in vitro Differentiation of Embryonic Stem Cells
Source: Front Cell Dev Biol. 2021 Apr 21;9:602197. doi: 10.3389/fcell.2021.602197 (PMC8097046; doi:10.3389/fcell.2021.602197)
Supplement: Supplementary file 4 [file Data_Sheet_1.docx]

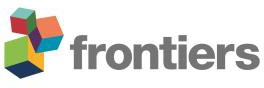


# Supplementary Methods

***Supplementary Material***

# Scrape loading/dye transfer assay

The SL/DT assay was performed as described. Proliferating CX26GJC-containing cells were grown for 7 days after transfer onto TRICs (feeder cells). Undifferentiated ESCs and TRICs were grown to confluency on dishes as a control. The medium was changed to HBSS plus 0.1% Lucifer yellow CH (L453, Invitrogen). Many parallel lines were cut into the dish with a razor blade, and after 15 min the cells were scrape-loaded with Lucifer yellow washed three times with HBSS and imaged. Scrape loading was quantified by measuring the distance from the scrape line to the point where the fluorescence intensity dropped to the background intensity.

# Western blot analysis

Cells were harvested and homogenized in ice-cold lysis buffer. Samples were subjected to 4-12% sodium dodecyl sulphate polyacrylamide gel electrophoresis (SDS-PAGE; Bio-Rad), followed by transfer to polyvinylidene difluoride membranes (Bio-Rad). The membranes were probed with rabbit anti-Smad3 (1:1000; Cell Signaling), rabbit anti-phospho Smad3 (1:1000; Cell Signaling), or mouse anti-β-actin (1:3000; Sigma) antibodies, and then with horseradish peroxidase-conjugated anti-rabbit IgG (1:15000; Sigma) or anti-mouse IgG (1:15000; Sigma). The bound antibodies were detected with an enhanced chemiluminescence detection system (Amersham).

Supplementary Material

# Supplementary Figures

A B

B

6 100

C

B

b

ab

a a

a a

B

ab ac

a

ab

a

b

CX26+ vesicles per aggregate

Percentage of aggregate containing CX26+ vesicle (%)

4

50

2

0 0


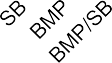

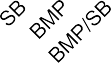

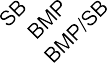

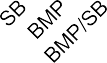


ESC iPSC ESC iPSC

**Supplementary Figure S1. The difference in induction efficiency to CX26+ cells between ESC and iPSC. (A)** The average number of CX26^+^ vesicles per aggregate and **(B)** percentage of aggregate containing CX26^+^ vesicles in different culture conditions. The data are expressed as the mean ± SE from four independently generated cell cultures per treatment; for each replicate, vesicles were quantified for six aggregates per treatment (n = 24 aggregates in total). Statistical differences among treatments were assessed by one-way ANOVA and Scheffe’s multiple comparison test. Different letters (a–c: P < 0.05; A-C: P < 0.01) represent significant differences.


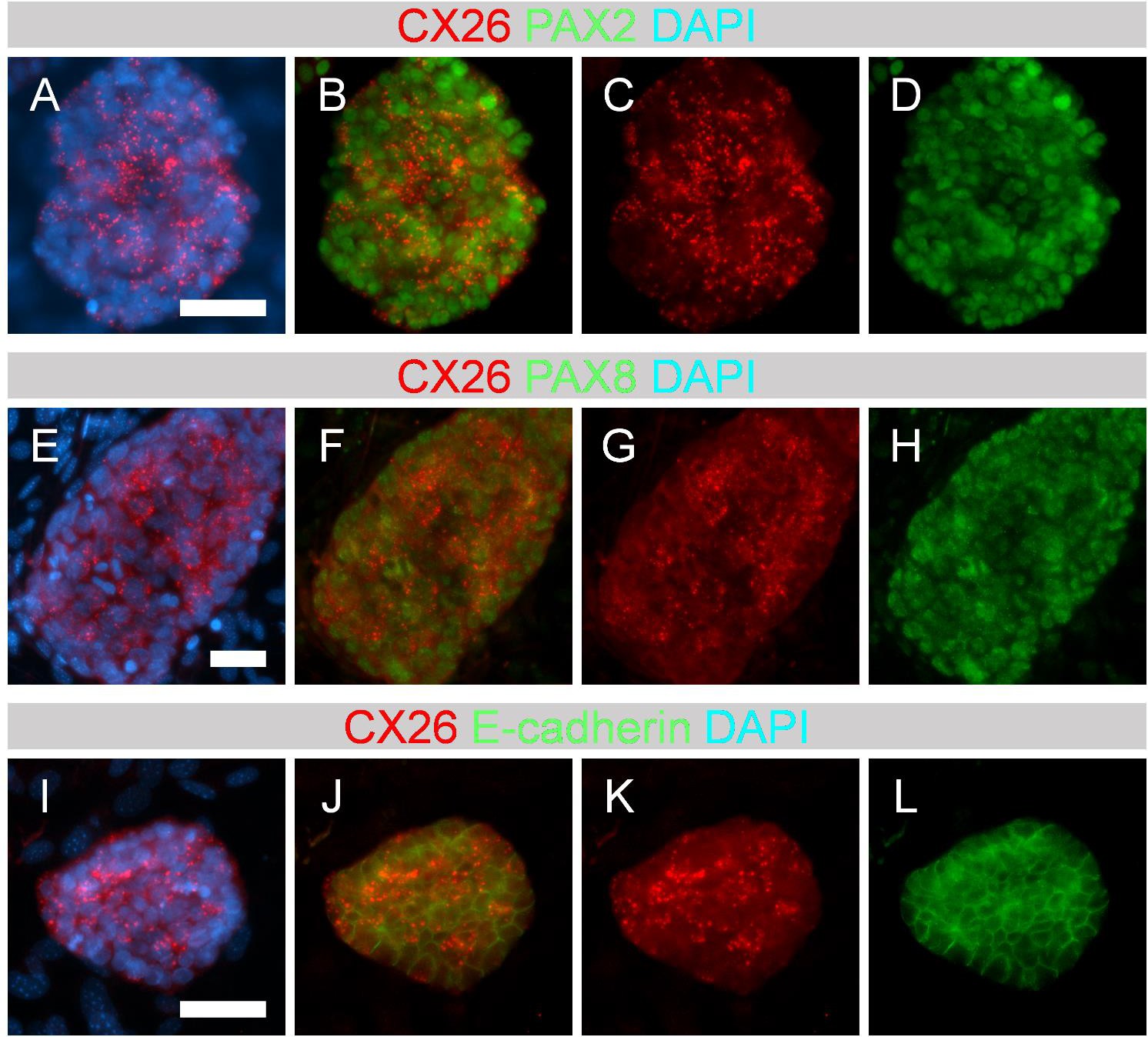


**Supplementary Figure S2. Characterization of CX26+ vesicles 3 days after being transferred onto TRIC feeder cells.** (**A**-**D**) staining for CX26 (red), PAX2 (green), and DAPI (blue). (**E**-**H**) staining for CX26 (red), PAX8 (green), and DAPI (blue). (**I**-**L**) staining for CX26 (red), E-cadherin (green), and DAPI (blue). Scale bars: 50 μm.

Supplementary Material


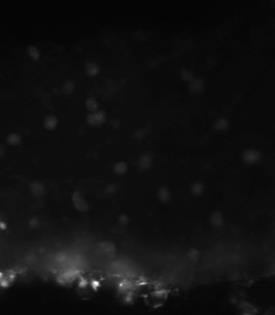

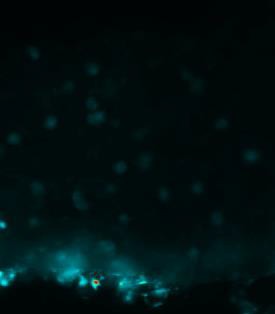

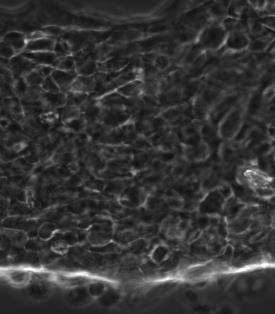


Undifferentiated

mESC

**B**

**E**

**H**


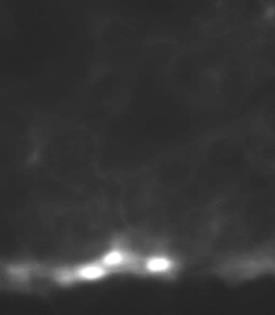

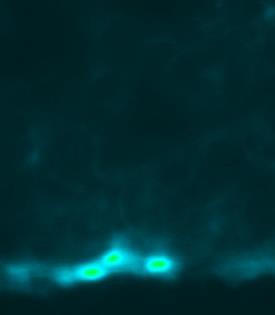

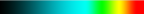

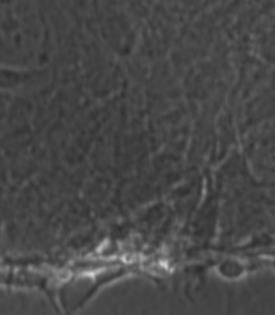


TRIC

feeder cell

**A**

**D**

0

Δf/f0

0.8

**G**


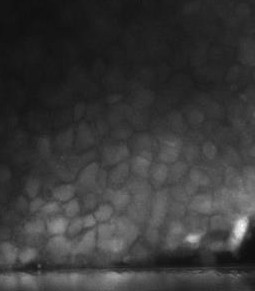

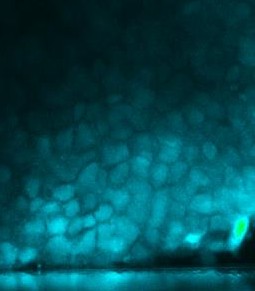

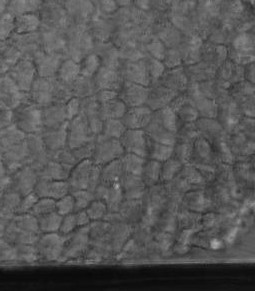


mESC derived

iCX26GJC

**C**

**F**

**I**

LY (Pseudo Color) Lucifer Yellow (LY)

# Supplementary Figure S3. The scrape loading-dye transfer assay revealed functional GJ formation in the mES-iCX26GJC.

PCM

Digital fluorescence images of cultured cells after scrape loading. The TRIC feeder cells **(A, D,** and **G)**, undifferentiated mESCs **(B, E,** and **H)**, and mESC-iCX26GJC at day 15 **(C, F,** and **I)**. **(A–C)** Dye transfer using LY. **(D–F)** Pseudocolor images indicating the range from low (black) to high (red) signal intensity of the images in **(A–C)**, respectively. **(G–I)** Phase contrast microscopy (PCM). Scale bars: 50 μm.


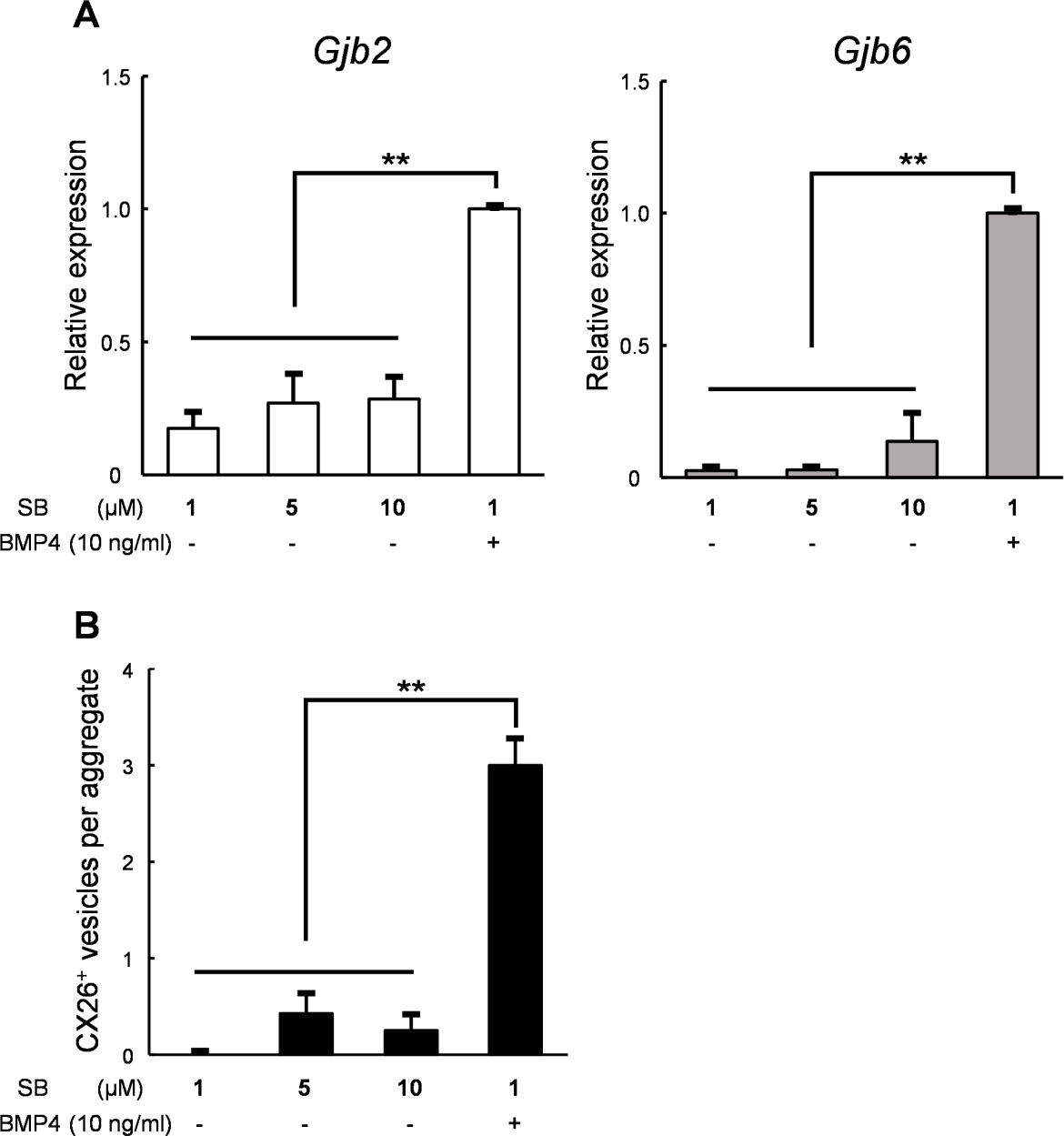


**Supplementary Figure S4. In SFEBq cultures, SB alone had less of an effect on *Gjb2*/*Gjb6* mRNA expression and the number of CX26+ vesicles as compared with BMP4 plus SB. (A)** Relative expression of *Gjb2* and *Gjb6* mRNA in day 7 aggregates from SFEBq cultures after treatment with the indicated concentration of SB (1, 5, or 10 μM) or with 1 μM SB and 10 ng/ml BMP4 (BMP4/1 μM SB). mRNA expression was normalized to that of cultures treated with BMP4/1 μM SB. The data are expressed as the mean ± SE from five independently generated cell cultures per treatment; for each replicate, expression was assessed in eight aggregates per treatment. **(B)** The average number of CX26^+^ vesicles per aggregate from aggregates treated as described in A. The data are expressed as the mean

± SE from three independently generated cell cultures per treatment; for each replicate, vesicles were quantified for 2–3 aggregates per treatment (n = 8 aggregates in total). Statistical differences among samples were assessed by a one-way ANOVA and Scheffe’s multiple comparison test; **p < 0.01.

Supplementary Material

# P-Smad3 β-actin Smad3 β-actin

**SB (μM)**

**BMP4 (10 ng/ml)**


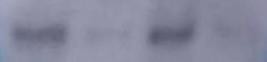

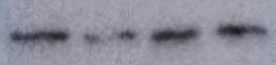

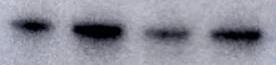

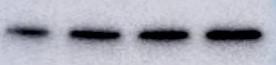


# 0 1 0 1

**- - + +**

# Supplementary Figure S5. SB431542 inhibited Activin/Nodal/TGF‐β pathway in ESC- iCX26GJC induction.

Smad3, and P-Smad3 were detected by Western blot analysis. β-Actin was detected as the loading control. P-Smad3: phosphorylated Smad3; SB: SB431542; BMP4: Bone Morphogenetic Protein-4.

# Supplementary Videos

**Supplementary Video S1. A three-dimensional (3D) image of a CX26+ vesicle from a day 7 aggregate.** The 3D image was reconstructed from consecutive confocal slices of the CX26^+^ vesicle in Figure 4A. Confocal stacks showing CX26 (red), F-actin (green), and DAPI (blue) staining indicate CX26-GJP−forming cells within the clear small vesicle.

**Supplementary Video S2. A three-dimensional (3D) image of CX26-GJP−forming cells in a CX26+ vesicle.** The 3D image was reconstructed from consecutive confocal slices in Figure 4B. Confocal stacks showing CX26 (red), F-actin (green), and DAPI (blue) staining indicate that CX26 formed GJPs at the cell-cell border.

**Supplementary Video S3. A three-dimensional (3D) image of CX26-GJP−forming cells in adherent cultures at day 15.** The 3D image was reconstructed from consecutive confocal slices from Figure 5F, H, and J. Confocal stacks showing CX26 (red), CX30 (green), and DAPI (blue) staining indicate that CX26 and CX30 co-assemble to form GJPs at the cell-cell border.
